# Frequency of Urinary Tract Infection Among Patients Undergoing Implant Fixation for Acute Trauma

**DOI:** 10.7759/cureus.49817

**Published:** 2023-12-02

**Authors:** Malik Amna Khatoon, Syed Muhammad Khalid Karim, Muhammad Wasim, Rufina Ali, Mariam Zaighum, Naveed Iqbal

**Affiliations:** 1 Orthopaedic Surgery, Dow University of Health Sciences, Dow International Medical College, Karachi, PAK; 2 Orthopaedics and Trauma, Dow University of Health Sciences, Dow International Medical College, Karachi, PAK; 3 Trauma and Orthopaedics, Shaheed Mohtarma Benazir Bhutto Institute of Trauma (SMBBIT), Karachi, PAK; 4 Orthopaedics and Trauma, Liaquat National Hospital and Medical College, Karachi, PAK

**Keywords:** intracapsular fracture, extracapsular hip fracture, neck of femur fractures, intertrochanteric hip fractures, implant fixation, antibiotic sensitivity, bacteria, urinary tract infection

## Abstract

Objective: This study aims to determine the frequency of urinary tract infection (UTI), identify the isolated bacteria, and assess antibiotic sensitivity in patients undergoing orthopedic implant fixation for hip fractures.

Methodology: After ethical approval from the institutional review board, this retrospective cross-sectional study was conducted at the Orthopedic Surgery Department of Dow University Hospital Karachi from June 2022 to June 2023. Through non-probability consecutive sampling, 186 patients above 16 years of age, of either gender, presenting with hip fractures such as intracapsular or extracapsular fractures, who underwent surgical fixation, were included in the study. A urine sample for urinalysis of these patients was sent on admission. Patients who presented with open fractures or those treated with conservative management were excluded from the study. The fracture diagnosis was confirmed on radiographs. All other relevant baseline investigations were also performed before surgery, per protocol, and urine-detailed and cultured reports were followed. In addition, each patient was asked about common symptoms of UTI before surgery and then diagnosed with UTI on positive urine culture and sensitivity (CS).

Results: Out of 186 hip fracture patients, 98 (52.7%) were males and 88 (47.3%) were females, with a mean age of 61.03 ± 16.43 (16-96) years. Pre-operative UTI symptoms were reported by 79 patients, including dysuria (16; 20.3%), polyuria (19; 24.0%), and burning (44; 55.7%). UTI was diagnosed on culture and sensitivity report in 65 (34.9%) patients with *Escherichia coli* as commonly diagnosed bacteria 35 (53.8%), followed by Enterococcus 8 (12.4%), Klebsiella 7 (10.9%), *Pseudomonas aeruginosa* 3 (4.7%), and Acinetobacter 2 (3.1%) patients. *E. coli* was sensitive to amikacin, amoxicillin/clavulanic acid, ampicillin, cefixime, ceftriaxone, cefuroxime, ciprofloxacin, colistin, cotrimoxazole, fosfomycin, gentamycin, levofloxacin, meropenem, nitrofurantoin, polymyxin B, and piperacillin-tazobactam.

Conclusion: Urinary tract infection is common in patients undergoing orthopedic implant fixation for hip fractures, which can lead to potentially serious outcomes. Overall, hygiene, prompt treatment, and standard protocol should be utilized to treat those infected and minimize the spread.

## Introduction

Surgical site infection (SSI) is a significant and detrimental consequence observed in patients with hip fractures. Studies have found a one-year mortality rate of over 50% in individuals who develop SSI, whereas those without SSI have a mortality rate of approximately 30% [1]. According to the surveillance report for the 2021-2022 period by the UK Health Security Agency, it was observed that the SSI rate for total hip replacement surgery in NHS (National Health Service) hospitals in England was 0.5% for cases involving acute trauma and chronic elective indications.

In contrast, the SSI rate for surgeries involving the repair of a neck or femur fracture, which included hip hemiarthroplasty and fracture fixation, was 0.8%. Based on the surveillance report for the period of 2021-2022, by the UK Health Security Agency, it was seen that Enterobacterales were the predominant causative organisms for SSIs across various surgical specialties. Furthermore, there has been an upward trend in their prevalence over the past ten years [2]. *Escherichia coli* was identified as the predominant species within the Enterobacterales order. The occurrence of SSIs caused by Enterobacterales was found to be 18.3% for superficial infections and 20.3% for deep infections in patients who underwent hip replacement surgery. Additionally, 55% of deep polymicrobial infections following surgery to repair a fractured neck of the femur featured Gram-negative bacteria. Given that Enterobacterales are the primary etiological agents responsible for urinary tract infection (UTI), there exists a legitimate concern regarding the potential association between UTI and SSI after hip fracture surgery [3].

The understanding of the relationship between UTIs and SSIs in individuals with hip fractures is limited. At present, there is a lack of agreement regarding the necessity of screening for and treating UTIs in individuals with hip fractures [4]. Patients who experience hip fractures tend to be of advanced age and have a higher prevalence of underlying medical conditions in comparison to individuals undergoing elective hip surgery [5]. This demographic profile renders them more vulnerable to the adverse consequences associated with SSI [6]. While it may be possible to postpone elective arthroplasty in cases where symptomatic bacteriuria is present, it is crucial to promptly perform surgical intervention for hip fractures. Ideally, this intervention should occur within 36 hours of the injury, as prolonged delays have been linked to increased death rates.

Therefore, it is impractical to delay the assessment of urine culture results and the resolution of urinary tract infections in patients with hip fractures. Additionally, UTIs may occur following hip fracture surgery. Urinary catheterization is commonly employed in individuals with hip fractures as a means to prevent or manage urinary retention, accommodate the restricted mobility of patients, and enable the monitoring of urine flow. Urinary catheterization poses a potential risk for UTIs due to the catheter's potential to serve as a site for bacterial colonization [7]. However, there is currently no consensus on the correlation between urinary catheterization (whether inserted before or following the occurrence of a hip fracture) and the risk of SSI in patients with hip fractures.

A limited number of studies have indicated the need to identify UTIs in patients undergoing orthopedic surgery to prevent surgical failure. Having said that, there is still a lack of well-documented data regarding the occurrence of UTIs specifically related to orthopedic implant fixation [8]. Hence, the present study was conducted in a tertiary care facility in Karachi to ascertain the frequency of UTI, identify the isolated bacteria, and assess antibiotic sensitivity in patients undergoing orthopedic implant fixation for hip fractures.

## Materials and methods

After ethical approval from the Institutional Review Board of Dow University of Health Sciences, this retrospective cross-sectional study was conducted at the Orthopedic Surgery Department of Dow University Hospital Karachi from June 2022 to June 2023. Through non-probability consecutive sampling, 186 patients above 16 years of age, of either gender, with hip fractures, intracapsular or extracapsular, who underwent surgical fixation were included in the study. Extracapsular hip fractures include those that occur below the hip joint capsule, such as intertrochanteric and subtrochanteric fractures. Intracapsular hip fractures are those that occur at the hip joint capsule, including femoral head and femoral neck fractures. All fractures were surgically managed, with internal fixation in intracapsular fractures and arthroplasty in extracapsular fractures.

Patients refusing surgical intervention or those with open fractures were excluded from the study. Each patient was evaluated for demographic data, medical history, any comorbidities, and pre-operative symptoms of UTI such as dysuria, polyuria, and burning. A clinical examination of each patient was performed for the identification of fractures. The diagnosis of fracture was confirmed based on radiological examination, including X-rays and computed tomography (CT).

Each patient was informed about the fracture and its surgical management (internal fixation or arthroplasty). A urine analysis of each patient was sent along with pre-operative labs. A midstream sample of urine was sent, and urinary catheterization of the patient was done if needed. The detailed urine report was indicated as positive if bacteria were present in the sample, and then the diagnosis of UTI was confirmed based on a positive urine culture and sensitivity (CS). The collected results were interpreted with Statistical Package for Social Sciences (SPSS) version 21.0 (IBM, Inc., Armonk, NY, USA). Data presented in tables are shown as mean ± standard deviation for continuous variables and frequency and percentages for categorical variables. Stratification was done with the outcome by applying a chi-square test with a significant p-value of ≤0.05.

## Results

In terms of the clinical and demographic parameters of the study participants, a total of 186 hip fracture patients were evaluated, out of which 98 (52.7%) were male and 88 (47.3%) were female (Table 1). The mean age of selected patients was 61.03 ± 16.43 (16-96) years. Most of the patients, 54 (29.0%), were suffering from diabetes mellitus (DM), followed by hypertension (HTN) in 23 (12.4%) patients, chronic kidney disease (CKD) in 9 (4.8%) patients, ischemic heart disease (IHD) in 7 (3.8%) patients, and dementia in only 1 (0.5%) patient.

**Table 1:** Demographic and clinical parameters of study participants *Mean± SD (standard deviation)

Variables	Frequency (n=186)
Gender	
Male, n (%)	88 (47.3%)
Female, n (%)	98 (52.7%)
Age (years)	61.03 ± 16.43*
16–30, n (%)	11 (5.9%)
31–45, n (%)	19 (10.2%)
46–60, n (%)	55 (29.6%)
61–75, n (%)	61 (32.8%)
76–90, n (%)	37 (19.9%)
>90, n (%)	3 (1.6%)
Comorbidities	
Diabetes mellitus, n (%)	54 (29.0%)
Hypertension, n (%)	23 (12.4%)
Chronic kidney disease, n (%)	9 (4.8%)
Ischemic heart disease, n (%)	7 (3.8%)
Dementia, n (%)	1 (0.5%)

The site of fracture was right in 115 (61.8%) patients and left in 71 (38.2%) patients (Table 2). The location of the fracture was intracapsular in 69 (37.1%) patients and extracapsular in 117 (62.9%) patients, and the same values are indicated with the operative procedure performed for each type of fracture, respectively. Fracture was displaced in 126 (67.7%) patients and non-displaced in 60 (32.3%) patients.

**Table 2:** Fracture characteristics of the study participants

Fracture characteristics	Frequency (n=186)
Site of fracture	
Right, n (%)	115 (61.8%)
Left, n (%)	71 (38.2%)
Location of fracture	
Intracapsular, n (%)	69 (37.1%)
Extracapsular, n (%)	117 (62.9%)
Characteristic of fracture	
Displaced, n (%)	126 (67.7%)
Non-displaced, n (%)	60 (32.3%)
Surgical management method	
Arthroplasty, n (%)	69 (37.1%)
Internal fixation, n (%)	117 (62.9%)

UTI symptoms were reported by 79 patients, including dysuria in 16 (20.3%), polyuria in 19 (24.0%), and burning in 44 (55.7%) patients (Table 3).

**Table 3:** Patients with urinary tract infection symptoms

Symptoms	Frequency (n=79)
Dysuria, n (%)	16 (20.3%)
Polyuria, n (%)	19 (24.0%)
Burning, n (%)	44 (55.7%)

Based on the detailed urine report, UTI was positive in 113 (60.8%) patients, but the final diagnosis of UTI was confirmed on urine CS, which was positive in 65 (34.9%) patients (Table 4). Isolated bacteria on urine CS were *Escherichia coli* in 35 (53.8%) patients, Enterococcus in 8 (12.4%) patients, Klebsiella in 7 (10.9%) patients, *Pseudomonas aeruginosa* in 3 (4.7%) patients, Acinetobacter in 2 (3.1%) patients, *Candida albicans* in 1 (1.5%) patient, *Candida glabrata* in 1 (1.5%) patient, *Staphylococcus aureus* in 1 (1.5%) patient, Streptococcus group D in 1 (1.5%) patient, *E. coli* + Enterococcus in (1 1.5%) patient, Acinetobacter + *P. aeruginosa* in 1 (1.5%) patient, Acinetobacter + Klebsiella in 1 (1.5%) patient and Enterococcus + *C. albicans* in 1 (1.5%) patient. There was insignificant bacterial growth in 2 (3.1%) patients, which was unable to show microorganisms or sensitivity, but urine culture was still deemed positive.

**Table 4:** Urinary tract infection diagnostic test

Diagnostic test	Frequency (n= 186)
Urine DR	
Positive, n (%)	113 (60.8%)
Negative, n (%)	73 (39.2%)
Urine CS	
Positive, n (%)	65 (34.9%)
Negative, n (%)	121 (65.1%)
Isolated microorganisms	Frequency (n= 65)
*E. coli*, n (%)	35 (53.8%)
Enterococcus, n (%)	8 (12.4%)
*C. albicans*, n (%)	1 (1.5%)
*P. aeruginosa*, n (%)	3 (4.7%)
Acinetobacter + *P. aeruginosa*, n (%)	1 (1.5%)
Acinetobacter + Klebsiella, n (%)	1 (1.5%)
Enterococcus + *C. albicans*, n (%)	1 (1.5%)
Klebsiella, n (%)	7 (10.9%)
Acinetobacter, n (%)	2 (3.1%)
*S. aureus*, n (%)	1 (1.5%)
Streptococcus group D, n (%)	1 (1.5%)
*C. glabrata*, n (%)	1 (1.5%)
*E. coli* + Enterococcus, n (%)	1 (1.5%)
Insignificant bacterial growth, n (%)	2 (3.1%)

The data were stratified according to patients' age, gender, and comorbidities (Table 5). A significant difference (p=0.002) was observed in the occurrence of UTI in females, while a significant difference (p=0.028) was observed in the occurrence of UTI in patients aged over 45 years. The occurrence of UTI was higher in patients with diabetes (p=0.003).

**Table 5:** Stratification of UTI with risk factors (n=186) *Significant p-value of ≤0.05

Risk factors	UTI		P-value
	Present (n=65)	Absent (n=121)	
Gender			
Male, n (%)	21 (32.3%)	67 (55.4%)	0.002*
Female, n (%)	44 (67.7%)	54 (44.6%)	
Age groups (Years)			
16–30, n (%)	2 (3.1%)	9 (7.4%)	0.028*
31–45, n (%)	2 (3.1%)	17 (14.0%)	
46–60, n (%)	21 (32.3%)	34 (28.1%)	
61–75, n (%)	25 (38.5%)	36 (29.8%)	
76–90, n (%)	13 (20.0%)	24 (19.8%)	
>90, n (%)	2 (3.1%)	1 (0.8%)	
Diabetes mellitus			
Yes, n (%)	28 (43.1%)	26 (21.5%)	0.003*
No, n (%)	37 (56.9%)	95 (78.5%)	
Hypertension			
Yes, n (%)	10 (15.4%)	13 (10.7%)	0.263
No, n (%)	55 (84.6%)	108 (89.3%)	
Ischemic heart disease			
Yes, n (%)	4 (6.2%)	3 (2.5%)	0.138
No, n (%)	61 (93.8%)	118 (97.5%)	
Chronic kidney disease			
Yes, n (%)	3 (4.6%)	6 (5.0%)	0.598
No, n (%)	62 (95.4%)	115 (95.0%)	
Dementia			
Yes, n (%)	1 (1.5%)	0 (0.0%)	0.349
No, n (%)	64 (98.5%)	121 (100.0%)	

Escherichia coli was sensitive to amikacin, amoxicillin/clavulanic acid, ampicillin, cefixime, ceftriaxone, cefuroxime, ciprofloxacin, colistin, cotrimoxazole, fosfomycin, gentamycin, levofloxacin, meropenem, nitrofurantoin, polymyxin B, and piperacillin-tazobactam. Enterococcus was sensitive to amoxicillin/clavulanic acid, ampicillin, fosfomycin, levofloxacin, nitrofurantoin, and vancomycin. Klebsiella was sensitive to amikacin, amoxicillin/clavulanic acid, cefixime, ceftriaxone, cefuroxime, ciprofloxacin, cotrimoxazole, fosfomycin, gentamycin, meropenem, nitrofurantoin, and piperacillin-tazobactam. *P. aeruginosa* was sensitive to amikacin, ceftazidime, ciprofloxacin, gentamycin, meropenem, and piperacillin-tazobactam. Acinetobacter was sensitive to amikacin, ceftriaxone, ciprofloxacin, cotrimoxazole, gentamycin, meropenem, and piperacillin-tazobactam (Table 6).

**Table 6:** Antibiotic sensitivity of isolated bacteria 0 (zero): no sensitivity of the antibiotic to the specific bacteria

Bacteria	Antibiotics sensitivity																		
	Amikacin	Amoxicillin/clavulanic acid	Ampicillin	Cefixime	Ceftazidime	Ceftriaxone	Cefuroxime	Ciprofloxacin	Cloxacillin	Colistin	Cotrimoxazole	Fosfomycin	Gentamycin	Levofloxacin	Meropenem	Nitrofurantoin	Polymyxin B	Piperacillin-tazobactam	Vancomycin
Acinetobacter	4	0	0	0	0	4	0	4	0	0	3	0	4	0	3	0	0	4	0
Escherichia coli	33	17	6	12	0	11	12	11	0	1	13	33	18	4	16	32	1	29	0
Enterococcus	0	7	7	0	0	0	0	0	0	0	0	9	0	5	0	6	0	0	2
Klebsiella	8	3	0	3	0	3	3	5	0	0	4	8	6	0	6	6	0	4	0
Pseudomonas aeruginosa	3	0	0	0	3	0	0	3	0	0	0	0	4	0	3	0	0	4	0
Staphylococcus aureus	1	0	0	0	0	0	0	1	1	0	0	0	1	0	0	1	0	0	1
Streptococcus group D	0	1	1	0	0	0	0	0	0	0	0	1	0	1	0	1	0	0	0

## Discussion

Infection is a common and significant complication frequently encountered by orthopedic specialists after orthopedic surgery, causing substantial harm and consequences. Despite advancements in medical tools, the use of pre-operative and postoperative antibiotics, and adherence to standard surgical procedures, infections remain a notable complication in orthopedic implant fixation. These infections not only lead to implant failure but also contribute to increased morbidity and mortality rates [9,10].

Urinary tract infection stands as the fourth leading cause of health-related infections, particularly in the context of an escalating infection risk among hospitalized patients. The reported incidence of UTIs linked to improper catheterization ranges from approximately 70% to 80%. Widely recognized as a significant risk factor for initiating and exacerbating UTIs, urinary catheterization plays a pivotal role in this scenario. UTIs, acquired within a hospital setting and caused by pathogenic bacteria, lead to the formation of bacteria reservoirs resistant to medications, culminating in elevated rates of illness and death [11,12].

Consequently, the present study aims to investigate the prevalence of UTIs among individuals undergoing orthopedic implant fixation treatment for hip fractures. Additionally, the research endeavors to pinpoint the specific pathogenic bacteria responsible for these infections and assess their susceptibility to various antibiotics.

The sample size in the current study reveals a noteworthy incidence of urinary tract infections (UTIs) among patients undergoing orthopedic implant fixation. In research conducted by Koulouvaris et al., the prevalence of postoperative UTIs in patients undergoing joint arthroplasty was documented at 6.9% [13]. Thakker et al. observed an initial frequency of 2.1% among orthopedic surgery patients, which significantly decreased to 1.1% after the implementation of a quality improvement strategy [12]. Additionally, Alvarez et al. reported a post-operative UTI rate of 1.1% in patients undergoing joint arthroplasty [14].

There is a significant disparity in the occurrence of UTIs in recent academic studies when compared to other developed nations [15]. This variation can be ascribed to differences in the use of cutting-edge medical and surgical instruments, the presence of advanced facilities in operating rooms, the availability of well-trained healthcare professionals, and strict adherence to established operating procedures and protocols.

As noted in the results, E. coli is the primarily isolated bacteria in the majority of the patients, followed by Enterococcus, Klebsiella, *P. aeruginosa*, and Acinetobacter. In the study led by Koulouvaris et al., it was observed that among UTI patients, 50.0% had *E. coli*, while the remaining 50.0% had Enterococcus [13]. Another investigation by Yassa et al. focusing on pre-operative UTIs found that *E. coli* was the most frequently identified bacterium, followed by *E. faecalis* and Pseudomonas [16]. The prevailing literature consistently emphasizes that *E. coli* stands out as the most identified bacterium in instances of urinary tract infections [17].

Our study highlights a significant link between UTIs and gender, revealing a statistically significant correlation, with the prevalence of UTIs notably higher in female patients. Koulouvaris et al. reported a distribution of 65.5% female patients and 34.5% male patients diagnosed with UTIs [13]. Numerous additional studies consistently establish that being female is a substantial risk factor for UTIs, with females experiencing a rate four times higher than males [14,18].

We also demonstrated an association between UTIs and various factors, including gender, diabetes mellitus, and urinary catheterization. A considerable percentage of female patients, diabetic patients, and those with urinary catheters were found to be affected by UTIs. In research conducted by Koulouvaris et al., 65.5% of the patients were female and 6.9% had urinary catheters, both groups experiencing UTIs [13]. Alvarez et al. observed that 72.2% of patients were female, 19.8% had diabetes, and all of them were diagnosed with UTIs [14]. Thakker et al. reported an initial urine catheterization rate of 55.2% among orthopedic surgery patients, which decreased to 19.8% after applying quality improvement efforts such as catheter use guidelines and education of front-line staff on catheter precautions [12]. Several studies consistently identify characteristics such as female gender, diabetes mellitus, and urinary catheterization as significant risk factors for UTIs, with the incidence in females being four times higher than in males [14-18].

In the context of orthopedic implant fixation, urinary catheterization poses a substantial risk for UTIs, especially in underdeveloped nations. Varied procedures for urine catheterization are employed in different Orthopedic settings due to the absence of widely accepted standardized criteria. Although routine catheterization is often recommended in orthopedic settings to reduce postoperative urinary retention, a significant risk factor for UTIs in patients undergoing orthopedic implant fixation [9,12,13], limited research has been conducted on reducing UTI prevalence by minimizing the use of urinary catheterization [14].

The approach to managing UTIs in individuals with hip fractures may differ depending on the particular clinical context and the patient's general health condition. The existence of a universally applicable procedure for screening and treating UTIs in hip fracture patients is lacking, as the appropriate method is contingent upon various aspects such as the patient's symptoms, medical history, and the guidelines established by the healthcare facility. Nevertheless, healthcare providers adhere to broad rules and principles while managing UTIs in this specific demographic.

Clinical assessments performed by healthcare workers evaluate patients for urinary tract infection symptoms. These symptoms may include increased urination frequency, urgency, burning, lower abdominal discomfort, and murky or malodorous urine. UTI symptoms may appear unnoticeable in the elderly, such as cognitive impairment, weariness, or worsening of pre-existing medical conditions.

Urinalysis, a frequent diagnostic test, detects bacteria, white blood cells, and other infection signs in urine. Positive urinalysis results and symptoms can support a urinary tract infection diagnosis. Sometimes a urine culture is ordered to identify the bacterial species causing the infection and choose the best treatment. However, urgent intervention is sometimes necessary, making the above step unnecessary. When a UTI is suspected and symptoms occur, healthcare providers usually start antibiotic therapy immediately. Antibiotics are chosen based on the patient's allergy history, prior antibiotic use, and local antibiotic resistance.

Hospitals and healthcare facilities adopt measures to reduce urinary tract infections. These measures usually include strict hygiene, catheter management, and timely movement, which reduce hip fracture patients' UTI risk. Recognizing that conditions vary, healthcare professionals use their clinical skills to choose the best course of action. Medical studies and standards evolve constantly. Thus, data and studies may change methods and practices over time.

The data obtained in this study is retrospective, so a limitation noted was that patients were not followed up after positive cultures, and we lacked the ability to determine if the subsequent cultures showed any growth after antibiotic therapy. A follow-up of patients would have also been beneficial to determine the correlation between positive urinary cultures and the presence of surgical site infection. Given the limited sample size, a large-scale and multi-center study would be beneficial in identifying more strains of urinary bacteria in hip fractures.

## Conclusions

Urinary tract infection is common in patients undergoing orthopedic implant fixation for hip fractures. Major risks are involved if intervention is not undertaken. Healthcare providers should choose treatment plans according to patients’ cultures and sensitivity reports. While managing urinary tract infections, healthcare facilities should set standard guidelines and take strict precautions to prevent the spread of bacteria.
